# Crystal structures of tetra­methyl­ammonium (2,2′-bi­pyridine)­tetra­cyanidoferrate(III) trihydrate and poly[[(2,2′-bi­pyridine-κ^2^ *N*,*N*′)di-μ_2_-cyanido-dicyanido(μ-ethyl­enedi­amine)(ethyl­enedi­amine-κ^2^ *N*,*N*′)­cadmium(II)iron(II)] monohydrate]

**DOI:** 10.1107/S2056989016006848

**Published:** 2016-04-29

**Authors:** Songwuit Chanthee, Wikorn Punyain, Supawadee Namuangrak, Kittipong Chainok

**Affiliations:** a Department of Chemistry, Faculty of Science and Research Center for Academic Excellence in Petroleum, Petrochemical and Advanced Materials, Naresuan University, Muang, Phitsanulok, 65000, Thailand; b National Nanotechnology Center, National Science and Technology Development Agency, Khlong Luang, Pathum Thani, 12120, Thailand; c Department of Physics, Faculty of Science and Technology, Thammasat University, Khlong Luang, Pathum Thani, 12120, Thailand

**Keywords:** crystal structure, cadmium, coordination polymers, cyanido complex, iron

## Abstract

The crystal structures of the building block tetra­methyl­ammonium (2,2′-bi­pyridine-κ^2^ *N*,*N*′)tetra­cyanidoferrate(III) trihydrate, [N(CH_3_)_4_][Fe(CN)_4_(C_10_H_8_N_2_)]·3H_2_O, (I), and a new two-dimensional cyanide-bridged bimetallic coordination polymer, poly[[(2,2′-bi­pyridine-κ^2^ *N*,*N*′)di-μ_2_-cyanido-dicyanido(μ-ethyl­enedi­amine-κ^2^ *N*:*N*′)(ethyl­enedi­amine-κ^2^ *N*,*N*′)cadmium(II)iron(II)] monohydrate], [CdFe(CN)_4_(C_10_H_8_N_2_)(C_2_H_8_N_2_)_2_]·H_2_O, (II), are reported. In the crystal of (I), pairs of [Fe(2,2′-bipy)(CN)_4_]^−^ units (2,2′-bipy is 2,2′-bi­pyri­dine) are linked together through π–π stacking between the pyridyl rings of the 2,2′-bipy ligands to form a graphite-like structure parallel to the *ab* plane. The three independent water mol­ecules are hydrogen-bonded alternately with each other, forming a ladder chain structure with *R*_4_^4^(8) and *R*_6_^6^(12) graph-set ring motifs, while the disordered [N(CH_3_)_4_]^+^ cations lie above and below the water chains, and the packing is stabilized by weak C—H⋯O hydrogen bonds. The water chains are further linked with adjacent sheets into a three-dimensional network *via* O—H⋯O hydrogen bonds involving the lattice water mol­ecules and the N atoms of terminal cyanide groups of the [Fe(2,2′-bipy)(CN)_4_]^−^ building blocks, forming an *R*_4_^4^(12) ring motif. Compound (II) features a two-dimensional {[Fe(2,2′-bipy)(CN)_4_Cd(en)_2_]}_*n*_ layer structure (en is ethyl­enedi­amine) extending parallel to (010) and constructed from {[Fe(2,2′-bipy)(CN)_4_Cd(en)]}_*n*_ chains inter­linked by bridging en ligands at the Cd atoms. Classical O—H⋯N and N—H⋯O hydrogen bonds involving the lattice water mol­ecule and N atoms of terminal cyanide groups and the N—H groups of the en ligands are observed within the layers. The layers are further connected *via* π–π stacking inter­actions between adjacent pyridine rings of the 2,2′-bipy ligands, completing a three-dimensional supra­molecular structure.

## Abstract 2

The cyanide complex [N(CH_3_)_4_][Fe(2,2′-bipy)(CN)_4_]·3H_2_O (2,2′-bipy is 2,2′-bi­pyridine) was synthesized as a building block for the construction of a new two-dimensional cyanide-bridged Fe–Cd bimetallic coordination polymer, [Fe(2,2′-bipy)(CN_4_)Cd(en)_2_]·H_2_O, in which ethyl­enedi­amine (en) adopts both bridging and chelating coordination modes.

## Chemical context   

Over the past several decades, hexa­cyanido­metallate anions, [*M*(CN)_6_]^*n*−^ (*n* = 2–4), have been used extensively as building blocks for the design and construction of a large number of high-dimensional cyanide-bridged bimetallic coordination polymers because of their ability to act as multidentate ligands to link numerous metal atoms through all six cyanide groups (Ohba & Ōkawa, 2000 ▸; Smith *et al.*, 2000 ▸; Berlinguette *et al.*, 2005 ▸). The highly insoluble three-dimensional Prussian blue and its more soluble Prussian blue analogues are perhaps the best known examples of this class of compounds, which are obtained by reacting the building block [*M*(CN)_6_]^3–^ with octa­hedrally coordinated transition metal ions (Buser *et al.*, 1977 ▸). The inclusion of a bidentate chelating ligand (*L*) such as 2,2′-bi­pyridine (2,2′-bipy) or 1,10-phenanthroline (1,10-phen) in cyanide-containing building blocks of general formula [*M*(*L*)(CN)_4_]^*n*−^ (*n* = 2, 3) instead of [*M*(CN)_6_]^*n*−^ has been a recent development in the field of low-dimensionality cyanide-bridged bimetallic coordination compounds (Lescouëzec *et al.*, 2001 ▸; Laza­rides *et al.*, 2007 ▸). The aromatic ligand *L* does not just block two coordination sites of the central atom, to yield one- and two-dimensional polymeric compounds, but also helps to stabilize the assembly as well as stabilizing the dimensionality of the three-dimensional supra­molecular structures through aromatic π–π stacking inter­actions (Lescouëzec *et al.*, 2002 ▸; Toma *et al.*, 2004 ▸). It is also known that the non-coordinating nitro­gen atoms of the cyanide groups can act as hydrogen-bond acceptors to self-assemble into various supra­molecular architectures (Xiang *et al.*, 2009 ▸). 
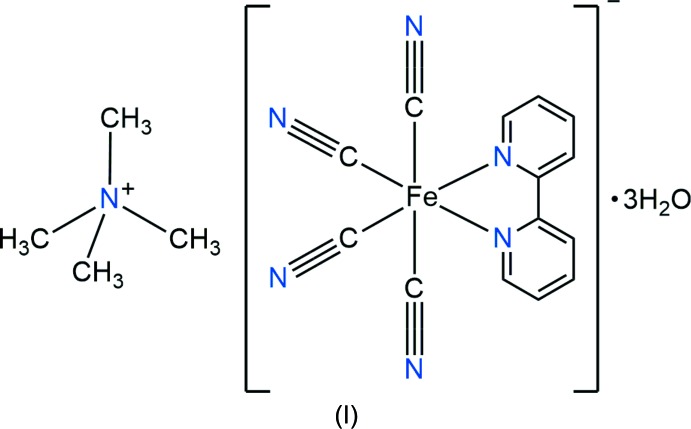
 
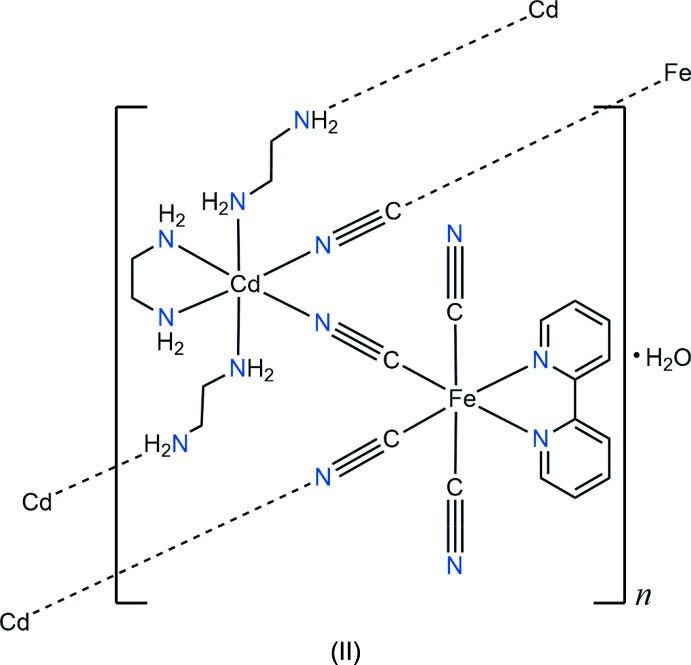


As part of our search for novel cyanide-bridged bimetallic coordination polymers, we herein describe the synthesis and crystal structure of [N(CH_3_)_4_][Fe(CN)_4_(C_10_H_8_N_2_)]·3H_2_O (I) building block and a new two-dimensional cyanide-bridged cadmium–iron(II) bimetallic coordination polymer, [CdFe(CN_4_)(C_10_H_8_N_2_)(C_2_H_8_N_2_)_2_]·H_2_O (II), in which ethylenedi­amine (en) adopts both bridging and chelating coordination modes.

## Structural commentary   

**Figure 1:**
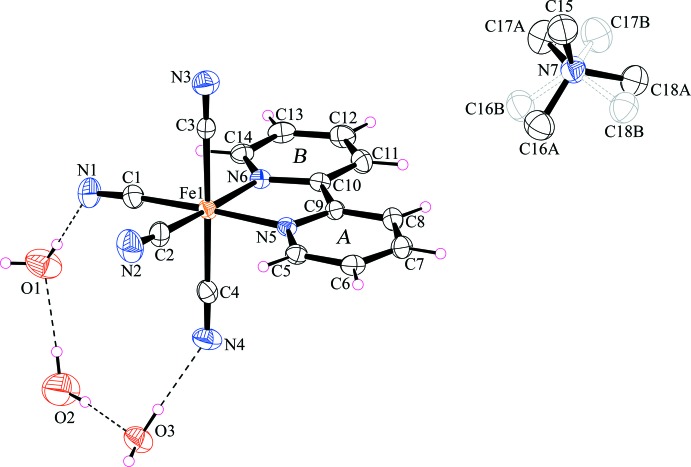
The asymmetric unit of (I), showing the atom-numbering scheme. Displacement ellipsoids are drawn at the 35% probability level. Dashed lines indicate O—H⋯O hydrogen bonds. Covalent bonds in the major and minor parts of the disordered are shaded differently and H atoms have been omitted for clarity. The labelling scheme *A* and *B* applied to the aromatic rings is used to identify the rings in the subsequent discussion.

The asymmetric unit of (I) consists of one [Fe(2,2′-bipy)(CN)_4_]^−^ anion, one disordered tetra­methyl­ammonium cation, [N(CH_3_)_4_]^+^ and three water mol­ecules, as displayed in Fig. 1 ▸. The Fe^III^ ion is coordinated by two nitro­gen atoms from one 2,2′-bipy ligand and four cyanide carbon atoms in a distorted octa­hedral geometry. This distortion around the metal atom is defined by the sum of the octa­hedral angular deviations from 90° (Σ), in which the trigonal distortion angle = 0 for a perfect octa­hedron (Marchivie *et al.*, 2005 ▸). In (I), Σ for twelve bond angles, *viz,* 5C—Fe—C, 6C—Fe—N and 1N—Fe—N, is 41.03°, confirming a distorted octa­hedral geometry around the central Fe^III^ ion. Another factor accounting for the distortion form ideal octa­hedral geometry of the Fe^III^ atom is the acute angle subtended by the chelating 2,2′-bipy ligand, *viz.* N5—Fe1—N6 = 81.14 (11)°. The three *trans* angles [*viz.* C1—Fe1—N5 = 175.01 (15), C2—Fe1—N6 = 175.52 (14) and C3—Fe1—C4 = 178.06 (15)°] are bent slightly from the ideal value of 180°. The iron atom and terminal cyanido groups, *viz.* [Fe1—C3≡N3 = 178.7 (3) and Fe1—C4≡N4 = 179.8 (4)°] are almost linear compared to the iron atom and the corresponding equatorial cyano groups [*viz.* Fe1—C1—N1 = 175.8 (4) and Fe1—C2—N2 = 176.6 (4)°]. This difference is probably caused by hydrogen bonding (see below). The Fe—C bond lengths range from 1.917 (4) to 1.969 (4) Å, whereas the Fe—N bond lengths are 1.981 (3) and 1.985 (3) Å. The whole mol­ecule of 2,2′-bipy ligand is planar with an r.m.s. deviation of 0.016 Å; the dihedral angle between the two pyridyl rings is 1.57 (18)°. Bond lengths and angles within the [Fe(2,2′-bipy)(CN)_4_]^−^ anion in (I) are in agreement with those reported for other cyanido and 2,2′-bipy-containing mononuclear iron(III) complexes such as K[Fe(2,2′-bipy)(CN)_4_]·H_2_O (Toma *et al.*, 2002 ▸), PPh_4_[Fe(2,2′-bipy)(CN)_4_]·H_2_O (Lescouëzec *et al.*, 2002 ▸) and AsPPh_4_[Fe(2,2′-bipy)(CN)_4_]·CH_3_CN (Toma *et al.*, 2007 ▸).

**Figure 2:**
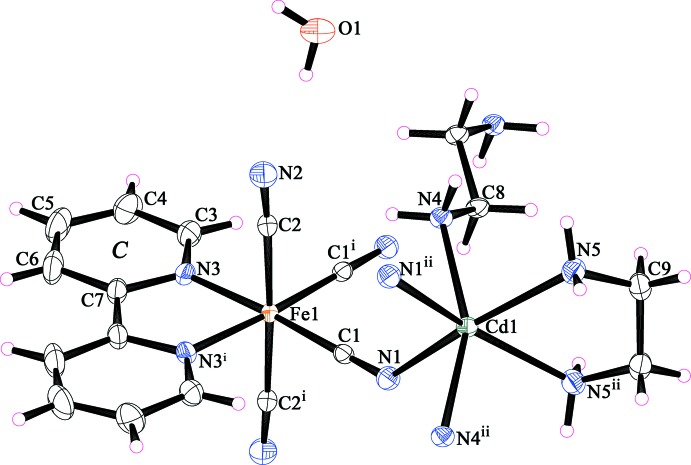
The structures of the molecular entities in (II), showing the atom-numbering scheme. Displacement ellipsoids are drawn at the 35% probability level. The pyridine ring labelled *C* is discussed in the text. [Symmetry codes: (i) 1 − *x*, *y*, 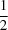 − *z*; (ii) −*x*, *y*, 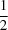 − *z*.]

**Figure 3:**
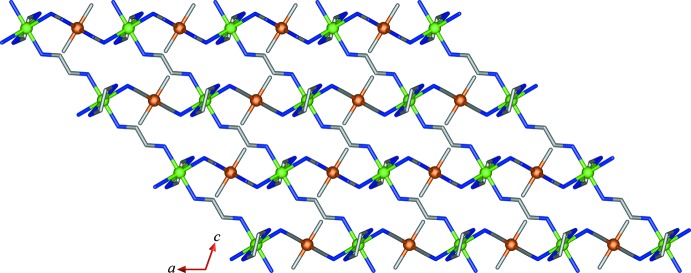
A view of the layer structure of (II) along the *b* axis. 2,2′-Bipy mol­ecules and H atoms bonded to C and N atoms of the en ligands have been omitted for clarity.

Compound (II) is a new cyanido-bridged Fe–Cd bimetallic coordination polymer synthesized using the precursor complex (I) as building block in which the Fe^III^ precursor was reduced to Fe^II^ under the crystallization conditions. The asymmetric unit contains half each of an [Fe(2,2′-bipy)(CN)_4_]^−^ anion and a [Cd(en)_2_]^2+^ cation, with the mol­ecules lying across twofold rotation axes, Fig. 2 ▸. The coordination polyhedron of Fe^II^ ion is a distorted octa­hedron with a Σ of 28.90°. The Fe—C—N angles for both bridging [Fe1—C1—N1 = 178.15 (14)°] and terminal [Fe1—C2—N2 = 176.85 (16)°] cyanide groups deviate slightly from strict linearity. The Fe—C_cyanide_ bond lengths at 1.8950 (16) and 1.9363 (17) Å are slightly shorter than the Fe—N_2,2′-bipy_ bond length, 1.9976 (14) Å. The Cd^II^ ion is six-coordinated by two N atoms from two cyanide groups, two N atoms from a chelating en ligand and two N atoms from two different bridging en ligands in a highly distorted octa­hedral geometry with a Σ of 108.08°. The Cd—N bond lengths and the N—Cd—N bond angles in (II) are in the range 2.3980 (15)–2.5046 (14) Å and 73.24 (5)–157.20 (5)°, respectively. These values are comparable to those observed in compounds (Et_4_N)[{Fe(CN)_6_}_3_{Cd(en)}_4_] (Maľarová *et al.*, 2003 ▸), [Fe(CN)_6_Cd(en)_2_] (Fu & Wang, 2005 ▸) and [{Fe(CN)_6_}_2_{Cd(en)}_3_]·4H_2_O (Maľarová *et al.*, 2006 ▸). Each [Fe(2,2′-bipy)(CN)_4_]^2–^ anion uses two cyanide groups to link [Cd(en)]^2+^cations, forming a chain of [Fe(2,2′-bipy)(CN)_4_Cd(en)] units running parallel to the *a* axis. Along the *b* axis, adjacent chains are then inter­connected through the N atoms of the bridging en ligands at the Cd atoms into a two-dimensional layer of [Fe(2,2′-bipy)(CN)_4_Cd(en)_2_], as shown in Fig. 3 ▸. The layer contains hexa­nuclear cyclic [{Fe(CN)_2_}_2_{Cd(en)}_2_] units with an Fe⋯Cd distance through the cyanide bridge and a Cd⋯Cd distance through the en bridge of 5.1292 (7) and 7.6692 (12) Å, respectively. The *M*⋯*M* distances across the cyclic windows vary from 5.5614 (10) to 14.0061 (10) Å.

## Supra­molecular features   

**Figure 4:**
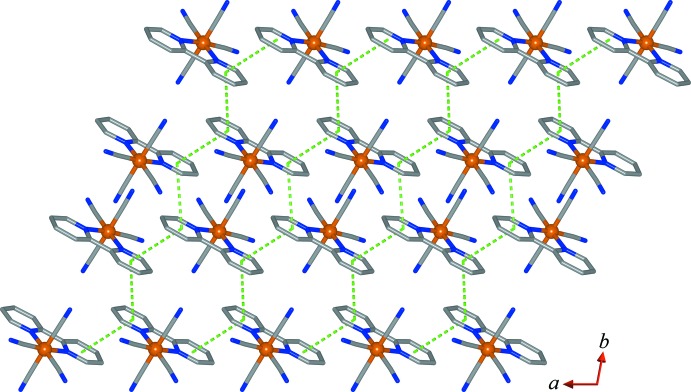
A view of the two-dimensional anionic [Fe(2,2′-bipy)(CN)_4_]^−^ graphite-like sheet structure in (I), parallel to the *ab* plane, with π–π inter­actions shown as dashed lines. H atoms have been omitted for clarity.

The three-dimensional supra­molecular structure in (I) is the result of combinations of inter­molecular inter­actions including aromatic π–π stacking and hydrogen bonds. As can be seen in Fig. 4 ▸, pairs of [Fe(2,2′-bipy)(CN)_4_]^−^ mol­ecules are linked together through the parallel pyridyl rings of the 2,2′-bipy ligands to generate a graphite-like layers parallel to the *ab* plane. Within the sheets, the neighbouring pyridyl moieties related by an inversion centre are in a head-to-head arrangement with centroid (*C*_g_) to centroid distances of 4.005 (3) Å [inter­planar angle = 0.0 (4)°] and 3.903 (3) Å [inter­planar angle = 0.0 (3)°] for rings *A*⋯*A* ^i^ and *B*⋯*B* ^ii^ [symmetry codes: (i) −*x*, 2 − *y*, 1 − *z*; (ii) 1 − *x*, 1 − *y*, 1 − *z*], respectively. The Fe^III^⋯Fe^III^ separations along the π–π stacking of parallel rings *A*⋯*A* ^i^ and rings *B*⋯*B* ^ii^ are 8.2821 (12) and 8.4572 (13) Å, respectively. The adjacent pyridyl rings *A* and *B* ^iii^ [symmetry code: (iii) *x* − 1, *y*, *z*] related by translation parallel to the *a* axis are arranged alternately in a head-to-tail manner with a *C*_g_⋯*C*_g_ distance of 3.865 (2) Å [inter­planar angle = 1.51 (12)°] and an Fe^III^⋯Fe^III^ separation of 6.8690 (9) Å.

**Figure 5:**
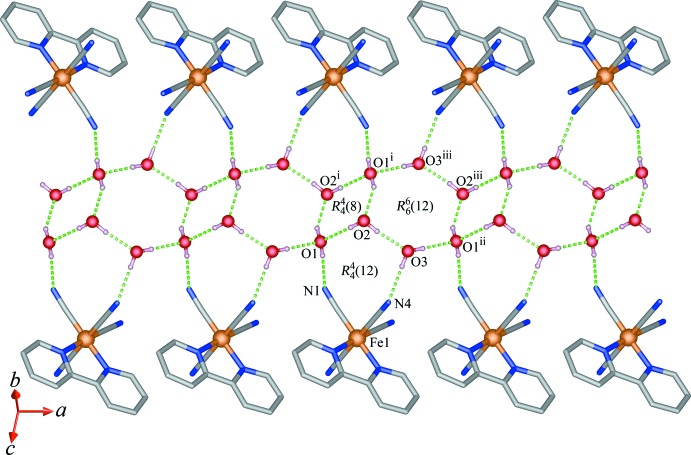
Self-assembly of the water tetra­mer (H_2_O)_4_ and hexa­mer (H_2_O)_6_ by O—H⋯O hydrogen bonds into the ladder-like chain, and representation of O—H⋯N hydrogen bonds between the water chain and anionic [Fe(2,2′-bipy)(CN)_4_]^−^ units. See Table 1 ▸ for symmetry codes.

A notable feature of (I) is the self-assembly of the tetra­meric (H_2_O)_4_ and hexa­meric (H_2_O)_6_ subunits into (H_2_O)_10_ units [the dihedral angle between the best plane of the (H_2_O)_4_ and (H_2_O)_6_ subunits is 55.2 (2)°]; neighbouring units are further joined together, giving rise to ladder-like water chains running parallel to the *a* axis. As can be seen from Fig. 5 ▸, the water mol­ecules at O1, O1^i^, O2, and O2^i^ (for symmetry code see Table 1 ▸) form centrosymmetric cyclic tetra­meric units through classical O—H⋯O hydrogen bonds with an 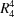
(8) ring motif according to graph-set notation. In this unit, each water monomer acts as a single donor and a single acceptor of hydrogen bonds, and the four water mol­ecules are perfectly coplanar (mean deviation of all non-hydrogen atoms = 0.00 Å). The average O⋯O distance in (I) is 2.805 Å. This value is comparable to the average distances for the gas-phase water tetra­mer (D_2_O)_4_ (2.78 Å; Liu *et al.*, 1996 ▸), liquid water (2.85 Å; Belch & Rice, 1987 ▸) and other tetra­meric water units in the solid state (2.81 Å; Tao *et al.*, 2004 ▸, and 2.83 Å; Long *et al.*, 2004 ▸). The average O⋯O⋯O angle is 90°, which is similar to those of the cyclic water tetra­mer found in liquid water and in the crystal host of metal–organic frameworks, [Cu(adipate)(4,4-bipy)]·2H_2_O (Long *et al.*, 2004 ▸) and [Cd_3_(pbtz)_3_(DMF)_4_(H_2_O)_2_]·4DMF·4H_2_O (Tao *et al.*, 2004 ▸).

The hexa­meric water unit has crystallographically imposed inversion symmetry. The six water mol­ecules O1^i^, O1^ii^, O2, O2^iii^, O3, and O3^iii^ (for symmetry codes, see Table 1 ▸) are almost coplanar with a mean deviation of 0.025 Å. Similar to the situation in the tetra­meric water unit, each water mol­ecule acts as both a single hydrogen-bond donor and acceptor, and is simultaneously involved in classical O—H⋯O inter­actions, leading to a cyclic 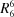
(12) hydrogen-bonding motif with an average O⋯O distance of 2.786 Å. This value is slightly shorter than the average distance for the tetra­meric unit and liquid water; however, it is comparable with the distance in ice *I*_h_ (2.74 Å; Eisenberg & Kauzmann, 1969 ▸) and water trapped in a metal–organic framework (2.78 Å; Ghosh & Bharadwaj, 2003 ▸). The average O⋯O⋯O angle in the planar hexa­meric unit is 120°, deviating considerably from the corresponding value of 109.3° in hexa­gonal ice (Fletcher, 1970 ▸). Another remarkable feature in (I) is that the ladder-like water chains are incorporated with the aromatic π–π stacking graphite-like layers through classical O—H⋯N hydrogen bonds involving the lattice water mol­ecules (O1 and O3) and the N atoms of the cyanido groups (N1 and N4), forming an 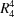
(12) ring motif. In addition, the [N(CH_3_)]^+^ cations lie above and below the water chains and take part in the formation of weak C—H⋯O hydrogen bonds with the water mol­ecule.

**Figure 6:**
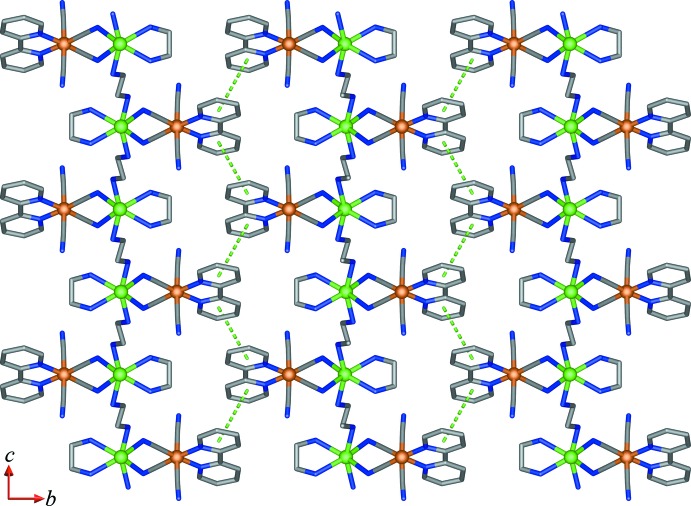
A portion of the crystal packing in (II) viewed in the *bc* plane showing π–π stacking inter­actions (dashed lines).

For (II), classical O—H⋯N and N—H⋯O hydrogen bonds involving the lattice water mol­ecules and N atoms of terminal cyanide groups and the N—H group of the en ligands are observed within a layer, Table 2 ▸. The layers are further linked together into a three-dimensional network *via* π–π stacking between adjacent pyridyl rings with *C*_g_⋯*C*_g_ distances of 4.2925 (18) [inter­planar angle = 1.55 (18)°] and 4.0642 (18) Å [inter­planar angle = 0.0 (3)°] for rings *C*⋯*C* ^iv^ and *C*⋯*C* ^v^ [symmetry codes: (iv) 2 − *x*, *y*, 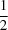 − *z*; (v) 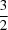 − *x*, 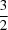 − *y*, 1 − *z*], respectively, Fig. 6 ▸.

## Synthesis and crystallization   

The building block N(CH_3_)_4_[Fe(2,2′-bipy)(CN)_4_]·3H_2_O (I) was prepared following the procedure described for PPh_4_[Fe(2,2′-bipy)(CN)_4_]·H_2_O (Lescouëzec *et al.*, 2002 ▸), except that tetra­methyl­ammonium chloride was used instead of tetra­phenyl­phospho­nium chloride. Dark-red single crystals of (I) suitable for structure determination were obtained by recrystallization from water and methanol (1:1, *v*/*v*). Analysis calculated for C_18_H_26_FeN_7_O: C, 48.66; H, 5.90; N, 22.07%. Found: C, 48.66; H, 5.90; N, 22.07%.

For the synthesis of (II), Cd(NO_3_)_2_·4H_2_O (0.062 g, 0.2 mmol) and ethyl­enedi­amine (stock solution, 0.01 ml, 0.2 mmol) were dissolved in distilled H_2_O (4 ml), and this was pipetted into one side of an H-tube. N(CH_3_)_4_[Fe(2,2′-bipy)(CN)_4_]·3H_2_O (0.089 g, 0.2 mmol) was dissolved in distilled H_2_O (4 ml), and this was pipetted into the other side arm of the H-tube. The H-tube (15 ml capacity) was then carefully filled with distilled H_2_O. Slow diffusion in the dark for three weeks yielded dark-yellow plate-shaped crystals of (II) suitable for X-ray crystallographic analysis. Analysis calculated for C_18_H_26_CdFeN_10_O: C, 38.15; H, 4.62; N, 24.72%. Found: C, 38.18; H, 4.60; N, 24.68%.

## Refinement   

Crystal data, data collection, and structure refinement details are summarized in Table 3 ▸. H atoms bonded to C and N atoms were placed at calculated positions and refined using a riding-model approximation, with C—H = 0.93 (aromatic), 0.96 (meth­yl) or 0.97 (methyl­ene) Å and N—H = 0.89 Å, and with *U*_iso_(H) = 1.5*U*_eq_(C) for methyl groups and 1.2*U*_eq_(C, N) otherwise. For (I), the water-H atoms were located in a difference Fourier map and refined with distance restraints: O—H = 0.84 (1) Å and H⋯H = 1.39 (2) Å with *U*_iso_(H) = 1.5*U*_eq_(O). For (II), the water-H atoms were refined with restraints of O—H = 0.82 (1) Å with *U*_iso_(H) = 1.5*U*_eq_(O). The tetra­metyl­ammonium cation in (I) exhibits rotational positional disorder in three of the methyl groups, and was refined with occupancy factors of 0.440 (6) for C16*A*, C17*A* and C18*A*, and 0.560 (6) for atoms C16*B*, C17*B*, and C18*B*. Anisotropic displacement parameters of all atoms were restrained using enhanced rigid-bond restraints (RIGU command, s.u.’s 0.001 Å^2^; Thorn *et al.*, 2012 ▸). The restraint SADI was also used for the disordered tetra­metyl­ammonium cation.

## Supplementary Material

Crystal structure: contains datablock(s) I, II. DOI: 10.1107/S2056989016006848/bg2584sup1.cif

Structure factors: contains datablock(s) I. DOI: 10.1107/S2056989016006848/bg2584Isup2.hkl

Structure factors: contains datablock(s) II. DOI: 10.1107/S2056989016006848/bg2584IIsup3.hkl

CCDC references: 1476008, 1476007

Additional supporting information:  crystallographic information; 3D view; checkCIF report

## Figures and Tables

**Table 1:** Hydrogen-bond geometry (Å, °) for (I)

*D*—H⋯*A*	*D*—H	H⋯*A*	*D*⋯*A*	*D*—H⋯*A*
C17*A*—H17*C*⋯O2^i^	0.96	2.50	3.112 (11)	122
O3—H3*A*⋯N4	0.84 (1)	2.00 (1)	2.841 (5)	178 (5)
O1—H1*A*⋯N1	0.84 (1)	2.03 (1)	2.859 (5)	176 (7)
O3—H3*B*⋯O1^ii^	0.85 (1)	1.89 (1)	2.736 (6)	174 (7)
O2—H2*A*⋯O3	0.84 (1)	1.87 (2)	2.709 (6)	172 (7)
O2—H2*B*⋯O1	0.84 (1)	1.98 (1)	2.818 (7)	177 (14)
O1—H1*B*⋯O2^iii^	0.84 (1)	2.02 (6)	2.792 (8)	152 (11)

Symmetry codes: (i) 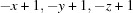; (ii) 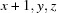; (iii) 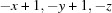.

**Table 2:** Hydrogen-bond geometry (Å, °) for (II)

*D*—H⋯*A*	*D*—H	H⋯*A*	*D*⋯*A*	*D*—H⋯*A*
N5—H5*A*⋯O1^i^	0.89	2.20	3.0726 (18)	167
O1—H1⋯N2	0.87 (1)	1.99 (1)	2.8045 (19)	156 (2)

Symmetry code: (i) 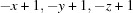.

**Table 3:** Experimental details

	(I)	(II)
Crystal data		
Chemical formula	(C_4_H_12_N)[Fe(CN)_4_(C_10_H_8_N_2_)]·3H_2_O	[CdFe(CN)_4_(C_10_H_8_N_2_)(C_2_H_8_N_2_)_2_]·H_2_O
*M*_r_	444.31	566.74
Crystal system, space group	Triclinic, *P* 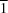	Monoclinic, *C*2/*c*
Temperature (K)	296	296
*a*, *b*, *c* (Å)	6.8690 (9), 11.9405 (16), 14.2731 (17)	7.4184 (14), 28.534 (5), 11.094 (2)
α, β, γ (°)	104.107 (4), 99.695 (4), 92.235 (4)	90, 109.143 (6), 90
*V* (Å^3^)	1115.2 (2)	2218.3 (7)
*Z*	2	4
Radiation type	Mo *K*α	Mo *K*α
μ (mm^−1^)	0.71	1.65
Crystal size (mm)	0.22 × 0.16 × 0.08	0.30 × 0.26 × 0.14

Data collection		
Diffractometer	Bruker APEXII D8 QUEST CMOS	Bruker APEXII D8 QUEST CMOS
Absorption correction	Multi-scan (*SADABS*; Bruker, 2014 ▸)	Multi-scan (*SADABS*; Bruker, 2014 ▸)
*T*_min_, *T*_max_	0.691, 0.745	0.633, 0.746
No. of measured, independent and observed [*I* > 2σ(*I*)] reflections	20120, 3982, 3015	51158, 2757, 2478
*R*_int_	0.072	0.038
(sin θ/λ)_max_ (Å^−1^)	0.599	0.667

Refinement		
*R*[*F* ^2^ > 2σ(*F* ^2^)], *wR*(*F* ^2^), *S*	0.053, 0.142, 1.04	0.020, 0.046, 1.07
No. of reflections	3982	2757
No. of parameters	321	146
No. of restraints	87	2
H-atom treatment	H atoms treated by a mixture of independent and constrained refinement	H atoms treated by a mixture of independent and constrained refinement
Δρ_max_, Δρ_min_ (e Å^−3^)	0.72, −0.59	0.47, −0.47

Computer programs: *APEX2* and *SAINT* (Bruker, 2014 ▸), *SHELXT* (Sheldrick, 2015*a* ▸), *SHELXL2014* (Sheldrick, 2015*b* ▸), *OLEX2* (Dolomanov *et al.*, 2009 ▸), *publCIF* (Westrip, 2010 ▸) and *enCIFer* (Allen *et al.*, 2004 ▸).

## supplementary crystallographic information

### (I) Tetramethylammonium (2,2'-bipyridine-κ2N,N')tetracyanidoferrate(III) trihydrate Crystal data

**Table:**

(C_4_H_12_N)[Fe(CN)_4_(C_10_H_8_N_2_)]·3H_2_O	*Z* = 2
*M**_r_* = 444.31	*F*(000) = 466
Triclinic, *P*1	*D*_x_ = 1.323 Mg m^−^^3^
*a* = 6.8690 (9) Å	Mo *K*α radiation, λ = 0.71073 Å
*b* = 11.9405 (16) Å	Cell parameters from 9941 reflections
*c* = 14.2731 (17) Å	θ = 3.0–36.4°
α = 104.107 (4)°	µ = 0.71 mm^−^^1^
β = 99.695 (4)°	*T* = 296 K
γ = 92.235 (4)°	Block, orange
*V* = 1115.2 (2) Å^3^	0.22 × 0.16 × 0.08 mm

### (I) Tetramethylammonium (2,2'-bipyridine-κ2N,N')tetracyanidoferrate(III) trihydrate Data collection

**Table:**

Bruker APEXII D8 QUEST CMOS diffractometer	3982 independent reflections
Radiation source: microfocus sealed x-ray tube, Incoatec Iµus	3015 reflections with *I* > 2σ(*I*)
GraphiteDouble Bounce Multilayer Mirror monochromator	*R*_int_ = 0.072
Detector resolution: 10.5 pixels mm^-1^	θ_max_ = 25.2°, θ_min_ = 3.0°
ω and φ scans	*h* = −8→8
Absorption correction: multi-scan (*SADABS*; Bruker, 2014)	*k* = −14→14
*T*_min_ = 0.691, *T*_max_ = 0.745	*l* = −16→17
20120 measured reflections	

### (I) Tetramethylammonium (2,2'-bipyridine-κ2N,N')tetracyanidoferrate(III) trihydrate Refinement

**Table:**

Refinement on *F*^2^	Primary atom site location: structure-invariant direct methods
Least-squares matrix: full	Hydrogen site location: mixed
*R*[*F*^2^ > 2σ(*F*^2^)] = 0.053	H atoms treated by a mixture of independent and constrained refinement
*wR*(*F*^2^) = 0.142	*w* = 1/[σ^2^(*F*_o_^2^) + (0.0712*P*)^2^ + 0.9213*P*] where *P* = (*F*_o_^2^ + 2*F*_c_^2^)/3
*S* = 1.04	(Δ/σ)_max_ < 0.001
3982 reflections	Δρ_max_ = 0.72 e Å^−^^3^
321 parameters	Δρ_min_ = −0.59 e Å^−^^3^
87 restraints	

### (I) Tetramethylammonium (2,2'-bipyridine-κ2N,N')tetracyanidoferrate(III) trihydrate Special details

**Table:**

Experimental. Absorption correction: SADABS-2014/4 (Bruker,2014/4) was used for absorption correction. wR2(int) was 0.0760 before and 0.0587 after correction. The Ratio of minimum to maximum transmission is 0.9266. The λ/2 correction factor is 0.00150.
Geometry. All esds (except the esd in the dihedral angle between two l.s. planes) are estimated using the full covariance matrix. The cell esds are taken into account individually in the estimation of esds in distances, angles and torsion angles; correlations between esds in cell parameters are only used when they are defined by crystal symmetry. An approximate (isotropic) treatment of cell esds is used for estimating esds involving l.s. planes.

### (I) Tetramethylammonium (2,2'-bipyridine-κ2N,N')tetracyanidoferrate(III) trihydrate Fractional atomic coordinates and isotropic or equivalent isotropic displacement parameters (Å^2^)

**Table:**

	*x*	*y*	*z*	*U*_iso_*/*U*_eq_	Occ. (<1)
Fe1	0.66815 (7)	0.18786 (4)	0.33212 (4)	0.03351 (19)	
N1	0.3698 (6)	0.2365 (4)	0.1661 (3)	0.0673 (11)	
N2	0.8300 (7)	0.0198 (4)	0.1707 (3)	0.0754 (12)	
N3	0.4210 (6)	−0.0265 (3)	0.3445 (3)	0.0589 (9)	
N4	0.9261 (6)	0.3973 (3)	0.3139 (3)	0.0596 (10)	
N5	0.8717 (4)	0.1745 (2)	0.4441 (2)	0.0316 (6)	
N6	0.5553 (4)	0.2840 (2)	0.4410 (2)	0.0329 (6)	
C1	0.4755 (6)	0.2152 (3)	0.2280 (3)	0.0458 (9)	
C2	0.7745 (6)	0.0849 (4)	0.2313 (3)	0.0467 (10)	
C3	0.5079 (5)	0.0512 (3)	0.3399 (2)	0.0353 (8)	
C4	0.8320 (6)	0.3212 (4)	0.3207 (3)	0.0415 (9)	
C5	1.0370 (5)	0.1194 (3)	0.4385 (3)	0.0368 (8)	
H5	1.0615	0.0823	0.3768	0.044*	
C6	1.1713 (5)	0.1153 (3)	0.5197 (3)	0.0420 (9)	
H6	1.2849	0.0764	0.5132	0.050*	
C7	1.1364 (6)	0.1694 (3)	0.6109 (3)	0.0456 (9)	
H7	1.2248	0.1668	0.6672	0.055*	
C8	0.9667 (6)	0.2285 (3)	0.6182 (3)	0.0449 (9)	
H8	0.9404	0.2664	0.6793	0.054*	
C9	0.8387 (5)	0.2298 (3)	0.5336 (3)	0.0341 (8)	
C10	0.6566 (5)	0.2913 (3)	0.5321 (3)	0.0346 (8)	
C11	0.5933 (6)	0.3533 (3)	0.6147 (3)	0.0460 (9)	
H11	0.6665	0.3583	0.6769	0.055*	
C12	0.4214 (6)	0.4078 (3)	0.6046 (3)	0.0495 (10)	
H12	0.3767	0.4498	0.6597	0.059*	
C13	0.3164 (6)	0.3992 (3)	0.5119 (3)	0.0458 (10)	
H13	0.1994	0.4353	0.5034	0.055*	
C14	0.3861 (5)	0.3368 (3)	0.4321 (3)	0.0388 (8)	
H14	0.3139	0.3308	0.3696	0.047*	
N7	0.7782 (5)	0.1799 (3)	0.9466 (2)	0.0597 (8)	
C15	0.7397 (8)	0.0530 (4)	0.9254 (4)	0.0789 (12)	
H15A	0.7621	0.0296	0.9859	0.118*	
H15B	0.8272	0.0161	0.8838	0.118*	
H15C	0.6048	0.0307	0.8928	0.118*	
C16A	0.901 (2)	0.1995 (11)	0.8753 (9)	0.078 (2)	0.440 (6)
H16A	0.8350	0.1608	0.8099	0.116*	0.440 (6)
H16B	1.0272	0.1693	0.8889	0.116*	0.440 (6)
H16C	0.9202	0.2811	0.8809	0.116*	0.440 (6)
C16B	0.7816 (19)	0.2208 (9)	0.8579 (6)	0.081 (2)	0.560 (6)
H16D	0.6484	0.2199	0.8237	0.121*	0.560 (6)
H16E	0.8551	0.1709	0.8158	0.121*	0.560 (6)
H16F	0.8436	0.2984	0.8760	0.121*	0.560 (6)
C17A	0.5779 (14)	0.2172 (11)	0.9193 (10)	0.085 (2)	0.440 (6)
H17A	0.4813	0.1690	0.9361	0.128*	0.440 (6)
H17B	0.5497	0.2105	0.8499	0.128*	0.440 (6)
H17C	0.5729	0.2964	0.9542	0.128*	0.440 (6)
C17B	0.6394 (16)	0.2374 (9)	1.0058 (8)	0.091 (2)	0.560 (6)
H17D	0.6423	0.3177	1.0052	0.137*	0.560 (6)
H17E	0.6766	0.2312	1.0721	0.137*	0.560 (6)
H17F	0.5079	0.2011	0.9789	0.137*	0.560 (6)
C18A	0.846 (2)	0.2542 (10)	1.0479 (6)	0.080 (2)	0.440 (6)
H18A	0.8136	0.3322	1.0500	0.120*	0.440 (6)
H18B	0.9863	0.2531	1.0664	0.120*	0.440 (6)
H18C	0.7803	0.2253	1.0927	0.120*	0.440 (6)
C18B	0.9780 (11)	0.1987 (8)	1.0119 (7)	0.0764 (19)	0.560 (6)
H18D	1.0251	0.2790	1.0273	0.115*	0.560 (6)
H18E	1.0690	0.1522	0.9787	0.115*	0.560 (6)
H18F	0.9678	0.1770	1.0715	0.115*	0.560 (6)
O3	1.0432 (6)	0.5315 (3)	0.1904 (3)	0.0675 (9)	
O1	0.3484 (7)	0.4452 (4)	0.1018 (4)	0.0881 (12)	
O2	0.6905 (8)	0.5827 (5)	0.1007 (4)	0.1151 (16)	
H3A	1.007 (7)	0.491 (3)	0.226 (3)	0.076 (16)*	
H1A	0.360 (10)	0.384 (3)	0.120 (4)	0.12 (2)*	
H3B	1.134 (9)	0.500 (6)	0.162 (5)	0.19 (4)*	
H2A	0.795 (6)	0.561 (6)	0.129 (5)	0.15 (3)*	
H2B	0.588 (7)	0.543 (9)	0.103 (9)	0.28 (7)*	
H1B	0.296 (16)	0.432 (6)	0.042 (2)	0.23 (6)*	

### (I) Tetramethylammonium (2,2'-bipyridine-κ2N,N')tetracyanidoferrate(III) trihydrate Atomic displacement parameters (Å^2^)

**Table:**

	*U*^11^	*U*^22^	*U*^33^	*U*^12^	*U*^13^	*U*^23^
Fe1	0.0330 (3)	0.0381 (3)	0.0315 (3)	0.0052 (2)	0.0066 (2)	0.0119 (2)
N1	0.064 (3)	0.084 (3)	0.055 (2)	0.008 (2)	−0.008 (2)	0.031 (2)
N2	0.082 (3)	0.089 (3)	0.053 (2)	0.024 (2)	0.025 (2)	0.003 (2)
N3	0.055 (2)	0.056 (2)	0.066 (2)	−0.0004 (19)	0.0092 (19)	0.0178 (19)
N4	0.054 (2)	0.060 (2)	0.077 (3)	0.0019 (19)	0.019 (2)	0.035 (2)
N5	0.0305 (15)	0.0323 (15)	0.0343 (15)	0.0044 (12)	0.0081 (13)	0.0111 (13)
N6	0.0304 (15)	0.0314 (16)	0.0389 (16)	0.0045 (12)	0.0072 (13)	0.0119 (13)
C1	0.047 (2)	0.051 (2)	0.041 (2)	0.0047 (19)	0.0058 (19)	0.0161 (19)
C2	0.048 (2)	0.053 (2)	0.036 (2)	0.0061 (19)	0.0045 (19)	0.0080 (19)
C3	0.0358 (19)	0.039 (2)	0.0297 (18)	0.0055 (17)	0.0033 (15)	0.0075 (16)
C4	0.038 (2)	0.052 (2)	0.038 (2)	0.0141 (18)	0.0082 (17)	0.0172 (18)
C5	0.0346 (19)	0.037 (2)	0.042 (2)	0.0053 (15)	0.0102 (16)	0.0131 (16)
C6	0.0337 (19)	0.041 (2)	0.057 (2)	0.0072 (16)	0.0088 (18)	0.0224 (19)
C7	0.041 (2)	0.053 (2)	0.046 (2)	0.0026 (18)	−0.0016 (18)	0.026 (2)
C8	0.045 (2)	0.054 (2)	0.037 (2)	0.0031 (18)	0.0056 (18)	0.0144 (18)
C9	0.0322 (18)	0.0364 (19)	0.0345 (18)	0.0009 (15)	0.0042 (15)	0.0121 (15)
C10	0.0330 (18)	0.0348 (19)	0.0382 (19)	0.0023 (15)	0.0096 (16)	0.0116 (16)
C11	0.050 (2)	0.050 (2)	0.037 (2)	0.0049 (19)	0.0113 (18)	0.0074 (18)
C12	0.048 (2)	0.046 (2)	0.054 (3)	0.0067 (19)	0.024 (2)	0.0024 (19)
C13	0.037 (2)	0.037 (2)	0.067 (3)	0.0083 (17)	0.017 (2)	0.0128 (19)
C14	0.0310 (18)	0.039 (2)	0.049 (2)	0.0039 (16)	0.0074 (17)	0.0156 (17)
N7	0.0690 (19)	0.0535 (17)	0.0545 (17)	0.0007 (15)	0.0077 (15)	0.0133 (14)
C15	0.089 (3)	0.0683 (19)	0.078 (3)	0.0004 (17)	0.014 (2)	0.0188 (17)
C16A	0.092 (4)	0.069 (4)	0.074 (4)	0.003 (3)	0.023 (3)	0.018 (3)
C16B	0.100 (5)	0.076 (4)	0.067 (3)	−0.001 (4)	0.010 (3)	0.024 (3)
C17A	0.084 (3)	0.085 (4)	0.079 (4)	0.012 (3)	0.006 (2)	0.012 (3)
C17B	0.100 (4)	0.097 (4)	0.081 (4)	0.024 (3)	0.024 (3)	0.023 (3)
C18A	0.086 (5)	0.081 (4)	0.065 (2)	−0.006 (3)	0.010 (2)	0.008 (2)
C18B	0.082 (3)	0.068 (4)	0.074 (3)	0.002 (2)	0.004 (2)	0.015 (3)
O3	0.075 (2)	0.068 (2)	0.070 (2)	0.0046 (18)	0.0206 (19)	0.0325 (18)
O1	0.083 (3)	0.089 (3)	0.116 (4)	0.025 (2)	0.035 (3)	0.058 (3)
O2	0.078 (3)	0.146 (4)	0.142 (4)	0.012 (3)	0.006 (3)	0.084 (4)

### (I) Tetramethylammonium (2,2'-bipyridine-κ2N,N')tetracyanidoferrate(III) trihydrate Geometric parameters (Å, º)

**Table:**

Fe1—N5	1.981 (3)	N7—C16B	1.468 (6)
Fe1—N6	1.985 (3)	N7—C17A	1.482 (7)
Fe1—C1	1.917 (4)	N7—C17B	1.460 (7)
Fe1—C2	1.917 (4)	N7—C18A	1.486 (7)
Fe1—C3	1.969 (4)	N7—C18B	1.498 (6)
Fe1—C4	1.969 (4)	C15—H15A	0.9600
N1—C1	1.132 (5)	C15—H15B	0.9600
N2—C2	1.142 (5)	C15—H15C	0.9600
N3—C3	1.105 (5)	C16A—H16A	0.9600
N4—C4	1.127 (5)	C16A—H16B	0.9600
N5—C5	1.339 (4)	C16A—H16C	0.9600
N5—C9	1.349 (4)	C16B—H16D	0.9600
N6—C10	1.348 (4)	C16B—H16E	0.9600
N6—C14	1.346 (4)	C16B—H16F	0.9600
C5—H5	0.9300	C17A—H17A	0.9600
C5—C6	1.367 (5)	C17A—H17B	0.9600
C6—H6	0.9300	C17A—H17C	0.9600
C6—C7	1.370 (5)	C17B—H17D	0.9600
C7—H7	0.9300	C17B—H17E	0.9600
C7—C8	1.391 (5)	C17B—H17F	0.9600
C8—H8	0.9300	C18A—H18A	0.9600
C8—C9	1.372 (5)	C18A—H18B	0.9600
C9—C10	1.475 (5)	C18A—H18C	0.9600
C10—C11	1.379 (5)	C18B—H18D	0.9600
C11—H11	0.9300	C18B—H18E	0.9600
C11—C12	1.373 (6)	C18B—H18F	0.9600
C12—H12	0.9300	O3—H3A	0.840 (10)
C12—C13	1.374 (6)	O3—H3B	0.847 (10)
C13—H13	0.9300	O1—H1A	0.836 (10)
C13—C14	1.371 (5)	O1—H1B	0.838 (10)
C14—H14	0.9300	O2—H2A	0.842 (10)
N7—C15	1.475 (5)	O2—H2B	0.840 (10)
N7—C16A	1.481 (7)		
			
N5—Fe1—N6	81.14 (11)	C15—N7—C17A	102.4 (6)
C1—Fe1—N5	175.01 (15)	C15—N7—C18A	122.6 (6)
C1—Fe1—N6	95.97 (14)	C15—N7—C18B	101.2 (5)
C1—Fe1—C2	86.42 (17)	C16A—N7—C17A	108.8 (8)
C1—Fe1—C3	92.47 (15)	C16A—N7—C18A	114.0 (8)
C1—Fe1—C4	87.33 (16)	C16B—N7—C15	112.9 (5)
C2—Fe1—N5	96.73 (14)	C16B—N7—C18B	111.8 (7)
C2—Fe1—N6	175.52 (14)	C17A—N7—C18A	102.4 (8)
C2—Fe1—C3	86.72 (16)	C17B—N7—C15	110.2 (6)
C2—Fe1—C4	91.34 (17)	C17B—N7—C16B	112.9 (7)
C3—Fe1—N5	91.57 (13)	C17B—N7—C18B	107.1 (6)
C3—Fe1—N6	89.39 (13)	N7—C15—H15A	109.5
C4—Fe1—N5	88.73 (13)	N7—C15—H15B	109.5
C4—Fe1—N6	92.55 (13)	N7—C15—H15C	109.5
C4—Fe1—C3	178.06 (15)	H15A—C15—H15B	109.5
C5—N5—Fe1	126.4 (2)	H15A—C15—H15C	109.5
C5—N5—C9	118.4 (3)	H15B—C15—H15C	109.5
C9—N5—Fe1	115.1 (2)	N7—C16A—H16A	109.5
C10—N6—Fe1	115.3 (2)	N7—C16A—H16B	109.5
C14—N6—Fe1	126.3 (3)	N7—C16A—H16C	109.5
C14—N6—C10	118.3 (3)	H16A—C16A—H16B	109.5
N1—C1—Fe1	175.8 (4)	H16A—C16A—H16C	109.5
N2—C2—Fe1	176.6 (4)	H16B—C16A—H16C	109.5
N3—C3—Fe1	178.7 (3)	N7—C16B—H16D	109.5
N4—C4—Fe1	179.8 (4)	N7—C16B—H16E	109.5
N5—C5—H5	118.7	N7—C16B—H16F	109.5
N5—C5—C6	122.7 (3)	H16D—C16B—H16E	109.5
C6—C5—H5	118.7	H16D—C16B—H16F	109.5
C5—C6—H6	120.5	H16E—C16B—H16F	109.5
C5—C6—C7	119.1 (3)	N7—C17A—H17A	109.5
C7—C6—H6	120.5	N7—C17A—H17B	109.5
C6—C7—H7	120.5	N7—C17A—H17C	109.5
C6—C7—C8	119.1 (4)	H17A—C17A—H17B	109.5
C8—C7—H7	120.5	H17A—C17A—H17C	109.5
C7—C8—H8	120.6	H17B—C17A—H17C	109.5
C9—C8—C7	118.8 (4)	N7—C17B—H17D	109.5
C9—C8—H8	120.6	N7—C17B—H17E	109.5
N5—C9—C8	121.9 (3)	N7—C17B—H17F	109.5
N5—C9—C10	114.4 (3)	H17D—C17B—H17E	109.5
C8—C9—C10	123.7 (3)	H17D—C17B—H17F	109.5
N6—C10—C9	114.0 (3)	H17E—C17B—H17F	109.5
N6—C10—C11	121.6 (3)	N7—C18A—H18A	109.5
C11—C10—C9	124.4 (3)	N7—C18A—H18B	109.5
C10—C11—H11	120.2	N7—C18A—H18C	109.5
C12—C11—C10	119.5 (4)	H18A—C18A—H18B	109.5
C12—C11—H11	120.2	H18A—C18A—H18C	109.5
C11—C12—H12	120.5	H18B—C18A—H18C	109.5
C11—C12—C13	119.0 (4)	N7—C18B—H18D	109.5
C13—C12—H12	120.5	N7—C18B—H18E	109.5
C12—C13—H13	120.4	N7—C18B—H18F	109.5
C14—C13—C12	119.2 (4)	H18D—C18B—H18E	109.5
C14—C13—H13	120.4	H18D—C18B—H18F	109.5
N6—C14—C13	122.4 (4)	H18E—C18B—H18F	109.5
N6—C14—H14	118.8	H3A—O3—H3B	109 (3)
C13—C14—H14	118.8	H1A—O1—H1B	112 (3)
C15—N7—C16A	105.4 (6)	H2A—O2—H2B	112 (3)
			
Fe1—N5—C5—C6	179.2 (3)	C6—C7—C8—C9	0.5 (6)
Fe1—N5—C9—C8	−179.9 (3)	C7—C8—C9—N5	0.8 (6)
Fe1—N5—C9—C10	−0.3 (4)	C7—C8—C9—C10	−178.8 (3)
Fe1—N6—C10—C9	2.4 (4)	C8—C9—C10—N6	178.2 (3)
Fe1—N6—C10—C11	−178.8 (3)	C8—C9—C10—C11	−0.5 (6)
Fe1—N6—C14—C13	178.2 (3)	C9—N5—C5—C6	1.2 (5)
N5—C5—C6—C7	0.1 (5)	C9—C10—C11—C12	179.7 (3)
N5—C9—C10—N6	−1.4 (4)	C10—N6—C14—C13	1.4 (5)
N5—C9—C10—C11	179.8 (3)	C10—C11—C12—C13	−0.1 (6)
N6—C10—C11—C12	1.0 (6)	C11—C12—C13—C14	−0.1 (6)
C5—N5—C9—C8	−1.7 (5)	C12—C13—C14—N6	−0.5 (6)
C5—N5—C9—C10	178.0 (3)	C14—N6—C10—C9	179.6 (3)
C5—C6—C7—C8	−1.0 (6)	C14—N6—C10—C11	−1.6 (5)

### (I) Tetramethylammonium (2,2'-bipyridine-κ2N,N')tetracyanidoferrate(III) trihydrate Hydrogen-bond geometry (Å, º)

**Table:**

*D*—H···*A*	*D*—H	H···*A*	*D*···*A*	*D*—H···*A*
C17*A*—H17*C*···O2^i^	0.96	2.50	3.112 (11)	122
O3—H3*A*···N4	0.84 (1)	2.00 (1)	2.841 (5)	178 (5)
O1—H1*A*···N1	0.84 (1)	2.03 (1)	2.859 (5)	176 (7)
O3—H3*B*···O1^ii^	0.85 (1)	1.89 (1)	2.736 (6)	174 (7)
O2—H2*A*···O3	0.84 (1)	1.87 (2)	2.709 (6)	172 (7)
O2—H2*B*···O1	0.84 (1)	1.98 (1)	2.818 (7)	177 (14)
O1—H1*B*···O2^iii^	0.84 (1)	2.02 (6)	2.792 (8)	152 (11)

Symmetry codes: (i) −*x*+1, −*y*+1, −*z*+1; (ii) *x*+1, *y*, *z*; (iii) −*x*+1, −*y*+1, −*z*.

### (II) Poly[[(2,2'-bipyridine-κ2N,N')di-µ2-cyanido-dicyanido(µ-ethylenediamine)(ethylenediamine-κ2N,N')cadmium(II)iron(II)] monohydrate] Crystal data

**Table:**

[CdFe(CN)_4_(C_10_H_8_N_2_)(C_2_H_8_N_2_)_2_]·H_2_O	*F*(000) = 1144
*M**_r_* = 566.74	*D*_x_ = 1.697 Mg m^−^^3^
Monoclinic, *C*2/*c*	Mo *K*α radiation, λ = 0.71073 Å
*a* = 7.4184 (14) Å	Cell parameters from 9748 reflections
*b* = 28.534 (5) Å	θ = 3.0–30.3°
*c* = 11.094 (2) Å	µ = 1.65 mm^−^^1^
β = 109.143 (6)°	*T* = 296 K
*V* = 2218.3 (7) Å^3^	Block, dark red
*Z* = 4	0.3 × 0.26 × 0.14 mm

### (II) Poly[[(2,2'-bipyridine-κ2N,N')di-µ2-cyanido-dicyanido(µ-ethylenediamine)(ethylenediamine-κ2N,N')cadmium(II)iron(II)] monohydrate] Data collection

**Table:**

Bruker APEXII D8 QUEST CMOS diffractometer	2757 independent reflections
Radiation source: microfocus sealed x-ray tube, Incoatec Iµus	2478 reflections with *I* > 2σ(*I*)
GraphiteDouble Bounce Multilayer Mirror monochromator	*R*_int_ = 0.038
Detector resolution: 10.5 pixels mm^-1^	θ_max_ = 28.3°, θ_min_ = 3.0°
φ and ω scans	*h* = −9→9
Absorption correction: multi-scan (*SADABS*; Bruker, 2014)	*k* = −38→37
*T*_min_ = 0.633, *T*_max_ = 0.746	*l* = −14→14
51158 measured reflections	

### (II) Poly[[(2,2'-bipyridine-κ2N,N')di-µ2-cyanido-dicyanido(µ-ethylenediamine)(ethylenediamine-κ2N,N')cadmium(II)iron(II)] monohydrate] Refinement

**Table:**

Refinement on *F*^2^	Primary atom site location: structure-invariant direct methods
Least-squares matrix: full	Hydrogen site location: mixed
*R*[*F*^2^ > 2σ(*F*^2^)] = 0.020	H atoms treated by a mixture of independent and constrained refinement
*wR*(*F*^2^) = 0.046	*w* = 1/[σ^2^(*F*_o_^2^) + (0.0207*P*)^2^ + 2.0817*P*] where *P* = (*F*_o_^2^ + 2*F*_c_^2^)/3
*S* = 1.07	(Δ/σ)_max_ = 0.004
2757 reflections	Δρ_max_ = 0.47 e Å^−^^3^
146 parameters	Δρ_min_ = −0.47 e Å^−^^3^
2 restraints	

### (II) Poly[[(2,2'-bipyridine-κ2N,N')di-µ2-cyanido-dicyanido(µ-ethylenediamine)(ethylenediamine-κ2N,N')cadmium(II)iron(II)] monohydrate] Special details

**Table:**

Experimental. SADABS-2014/5 (Bruker,2014/5) was used for absorption correction. wR2(int) was 0.0955 before and 0.0483 after correction. The Ratio of minimum to maximum transmission is 0.8480. The λ/2 correction factor is 0.00150.
Geometry. All esds (except the esd in the dihedral angle between two l.s. planes) are estimated using the full covariance matrix. The cell esds are taken into account individually in the estimation of esds in distances, angles and torsion angles; correlations between esds in cell parameters are only used when they are defined by crystal symmetry. An approximate (isotropic) treatment of cell esds is used for estimating esds involving l.s. planes.

### (II) Poly[[(2,2'-bipyridine-κ2N,N')di-µ2-cyanido-dicyanido(µ-ethylenediamine)(ethylenediamine-κ2N,N')cadmium(II)iron(II)] monohydrate] Fractional atomic coordinates and isotropic or equivalent isotropic displacement parameters (Å^2^)

**Table:**

	*x*	*y*	*z*	*U*_iso_*/*U*_eq_	
Cd1	1.0000	0.49884 (2)	0.7500	0.02579 (6)	
Fe1	0.5000	0.37467 (2)	0.7500	0.02273 (7)	
N4	0.75926 (17)	0.48296 (5)	0.56872 (12)	0.0267 (3)	
H4A	0.7937	0.4917	0.5024	0.032*	
H4B	0.7400	0.4521	0.5632	0.032*	
N3	0.3184 (2)	0.32132 (5)	0.68950 (13)	0.0302 (3)	
N1	0.8094 (2)	0.44919 (5)	0.85021 (13)	0.0322 (3)	
N5	0.9225 (2)	0.56629 (5)	0.85229 (14)	0.0366 (3)	
H5A	0.7964	0.5699	0.8271	0.044*	
H5B	0.9639	0.5621	0.9365	0.044*	
C8	0.5794 (2)	0.50634 (5)	0.56058 (14)	0.0268 (3)	
H8A	0.5983	0.5400	0.5636	0.032*	
H8B	0.5425	0.4975	0.6336	0.032*	
C1	0.6899 (2)	0.42120 (5)	0.81322 (14)	0.0247 (3)	
N2	0.5958 (3)	0.37056 (6)	0.49974 (16)	0.0510 (4)	
C2	0.5568 (2)	0.37319 (5)	0.59168 (15)	0.0304 (3)	
C7	0.3972 (3)	0.27812 (6)	0.71567 (17)	0.0359 (4)	
C3	0.1298 (3)	0.32437 (7)	0.62837 (18)	0.0397 (4)	
H3	0.0746	0.3539	0.6117	0.048*	
C4	0.0142 (3)	0.28557 (8)	0.5892 (2)	0.0524 (5)	
H4	−0.1158	0.2891	0.5466	0.063*	
C9	1.0114 (3)	0.60852 (6)	0.82011 (18)	0.0441 (4)	
H9A	1.1459	0.6092	0.8702	0.053*	
H9B	0.9517	0.6363	0.8406	0.053*	
C6	0.2860 (3)	0.23784 (7)	0.6780 (2)	0.0531 (5)	
H6	0.3422	0.2084	0.6963	0.064*	
C5	0.0941 (4)	0.24163 (8)	0.6142 (2)	0.0593 (6)	
H5	0.0193	0.2150	0.5883	0.071*	
O1	0.5000	0.40519 (9)	0.2500	0.0599 (6)	
H1	0.510 (5)	0.3877 (6)	0.3156 (11)	0.093 (10)*	

### (II) Poly[[(2,2'-bipyridine-κ2N,N')di-µ2-cyanido-dicyanido(µ-ethylenediamine)(ethylenediamine-κ2N,N')cadmium(II)iron(II)] monohydrate] Atomic displacement parameters (Å^2^)

**Table:**

	*U*^11^	*U*^22^	*U*^33^	*U*^12^	*U*^13^	*U*^23^
Cd1	0.01948 (8)	0.02751 (9)	0.02482 (9)	0.000	−0.00030 (6)	0.000
Fe1	0.02656 (15)	0.01792 (14)	0.02166 (14)	0.000	0.00513 (11)	0.000
N4	0.0199 (6)	0.0310 (7)	0.0259 (6)	0.0007 (5)	0.0030 (5)	−0.0003 (5)
N3	0.0388 (8)	0.0243 (6)	0.0276 (7)	−0.0063 (6)	0.0111 (6)	−0.0029 (5)
N1	0.0300 (7)	0.0325 (7)	0.0321 (7)	−0.0047 (6)	0.0074 (6)	0.0002 (6)
N5	0.0442 (9)	0.0326 (8)	0.0365 (8)	0.0031 (6)	0.0178 (7)	0.0032 (6)
C8	0.0190 (7)	0.0330 (8)	0.0239 (7)	0.0019 (6)	0.0009 (6)	−0.0024 (6)
C1	0.0270 (7)	0.0246 (7)	0.0214 (7)	0.0042 (6)	0.0066 (6)	0.0024 (6)
N2	0.0791 (13)	0.0434 (9)	0.0369 (9)	0.0018 (9)	0.0277 (9)	0.0007 (7)
C2	0.0373 (8)	0.0218 (7)	0.0292 (8)	−0.0003 (6)	0.0072 (7)	0.0009 (6)
C7	0.0507 (10)	0.0233 (8)	0.0370 (9)	−0.0040 (7)	0.0188 (8)	−0.0015 (7)
C3	0.0390 (10)	0.0344 (9)	0.0411 (10)	−0.0077 (8)	0.0069 (8)	−0.0013 (7)
C4	0.0465 (11)	0.0498 (12)	0.0560 (13)	−0.0194 (9)	0.0100 (10)	−0.0085 (10)
C9	0.0634 (13)	0.0291 (9)	0.0427 (11)	−0.0014 (8)	0.0213 (9)	−0.0030 (7)
C6	0.0699 (15)	0.0250 (9)	0.0675 (14)	−0.0094 (9)	0.0265 (12)	−0.0060 (9)
C5	0.0670 (15)	0.0395 (11)	0.0710 (15)	−0.0264 (10)	0.0222 (12)	−0.0154 (10)
O1	0.0733 (15)	0.0704 (15)	0.0342 (11)	0.000	0.0151 (11)	0.000

### (II) Poly[[(2,2'-bipyridine-κ2N,N')di-µ2-cyanido-dicyanido(µ-ethylenediamine)(ethylenediamine-κ2N,N')cadmium(II)iron(II)] monohydrate] Geometric parameters (Å, º)

**Table:**

Cd1—N4^i^	2.2546 (13)	N5—H5B	0.8900
Cd1—N4	2.2547 (13)	N5—C9	1.473 (2)
Cd1—N1^i^	2.5046 (14)	C8—C8^iii^	1.512 (3)
Cd1—N1	2.5045 (14)	C8—H8A	0.9700
Cd1—N5^i^	2.3981 (15)	C8—H8B	0.9700
Cd1—N5	2.3980 (15)	N2—C2	1.150 (2)
Fe1—N3	1.9976 (14)	C7—C7^ii^	1.465 (4)
Fe1—N3^ii^	1.9976 (14)	C7—C6	1.396 (3)
Fe1—C1^ii^	1.8951 (16)	C3—H3	0.9300
Fe1—C1	1.8950 (16)	C3—C4	1.380 (3)
Fe1—C2^ii^	1.9362 (17)	C4—H4	0.9300
Fe1—C2	1.9363 (17)	C4—C5	1.376 (3)
N4—H4A	0.8900	C9—C9^i^	1.509 (4)
N4—H4B	0.8900	C9—H9A	0.9700
N4—C8	1.4681 (19)	C9—H9B	0.9700
N3—C7	1.354 (2)	C6—H6	0.9300
N3—C3	1.343 (2)	C6—C5	1.370 (3)
N1—C1	1.163 (2)	C5—H5	0.9300
N5—H5A	0.8900	O1—H1	0.865 (9)
			
N4^i^—Cd1—N4	156.82 (7)	C3—N3—C7	118.18 (15)
N4^i^—Cd1—N1^i^	83.39 (5)	C1—N1—Cd1	135.03 (12)
N4^i^—Cd1—N1	83.56 (5)	Cd1—N5—H5A	109.6
N4—Cd1—N1	83.39 (5)	Cd1—N5—H5B	109.6
N4—Cd1—N1^i^	83.56 (5)	H5A—N5—H5B	108.1
N4^i^—Cd1—N5	88.96 (5)	C9—N5—Cd1	110.25 (11)
N4—Cd1—N5^i^	88.96 (5)	C9—N5—H5A	109.6
N4—Cd1—N5	109.92 (5)	C9—N5—H5B	109.6
N4^i^—Cd1—N5^i^	109.91 (5)	N4—C8—C8^iii^	111.91 (16)
N1—Cd1—N1^i^	111.12 (7)	N4—C8—H8A	109.2
N5—Cd1—N1^i^	157.20 (5)	N4—C8—H8B	109.2
N5^i^—Cd1—N1^i^	89.20 (5)	C8^iii^—C8—H8A	109.2
N5^i^—Cd1—N1	157.20 (5)	C8^iii^—C8—H8B	109.2
N5—Cd1—N1	89.20 (5)	H8A—C8—H8B	107.9
N5—Cd1—N5^i^	73.24 (7)	N1—C1—Fe1	178.15 (14)
N3—Fe1—N3^ii^	80.69 (8)	N2—C2—Fe1	176.85 (16)
C1^ii^—Fe1—N3	94.13 (6)	N3—C7—C7^ii^	114.47 (10)
C1—Fe1—N3^ii^	94.12 (6)	N3—C7—C6	120.93 (18)
C1^ii^—Fe1—N3^ii^	174.82 (6)	C6—C7—C7^ii^	124.60 (13)
C1—Fe1—N3	174.81 (6)	N3—C3—H3	118.5
C1—Fe1—C1^ii^	91.06 (9)	N3—C3—C4	122.95 (19)
C1^ii^—Fe1—C2	92.07 (6)	C4—C3—H3	118.5
C1—Fe1—C2	89.69 (7)	C3—C4—H4	120.5
C1^ii^—Fe1—C2^ii^	89.69 (7)	C5—C4—C3	119.0 (2)
C1—Fe1—C2^ii^	92.07 (7)	C5—C4—H4	120.5
C2^ii^—Fe1—N3^ii^	90.11 (6)	N5—C9—C9^i^	110.03 (14)
C2—Fe1—N3^ii^	87.98 (6)	N5—C9—H9A	109.7
C2—Fe1—N3	90.11 (6)	N5—C9—H9B	109.7
C2^ii^—Fe1—N3	87.98 (6)	C9^i^—C9—H9A	109.7
C2^ii^—Fe1—C2	177.49 (9)	C9^i^—C9—H9B	109.7
Cd1—N4—H4A	108.8	H9A—C9—H9B	108.2
Cd1—N4—H4B	108.8	C7—C6—H6	120.0
H4A—N4—H4B	107.7	C5—C6—C7	120.1 (2)
C8—N4—Cd1	113.68 (9)	C5—C6—H6	120.0
C8—N4—H4A	108.8	C4—C5—H5	120.6
C8—N4—H4B	108.8	C6—C5—C4	118.82 (19)
C7—N3—Fe1	115.19 (12)	C6—C5—H5	120.6
C3—N3—Fe1	126.62 (12)		
			
Cd1—N4—C8—C8^iii^	−178.38 (14)	C7—N3—C3—C4	−1.2 (3)
Cd1—N5—C9—C9^i^	43.1 (2)	C7^ii^—C7—C6—C5	179.9 (2)
Fe1—N3—C7—C7^ii^	0.1 (2)	C7—C6—C5—C4	−0.5 (4)
Fe1—N3—C7—C6	−179.70 (15)	C3—N3—C7—C7^ii^	−179.03 (18)
Fe1—N3—C3—C4	179.74 (15)	C3—N3—C7—C6	1.2 (3)
N3—C7—C6—C5	−0.3 (3)	C3—C4—C5—C6	0.4 (4)
N3—C3—C4—C5	0.4 (3)		

Symmetry codes: (i) −*x*+2, *y*, −*z*+3/2; (ii) −*x*+1, *y*, −*z*+3/2; (iii) −*x*+1, −*y*+1, −*z*+1.

### (II) Poly[[(2,2'-bipyridine-κ2N,N')di-µ2-cyanido-dicyanido(µ-ethylenediamine)(ethylenediamine-κ2N,N')cadmium(II)iron(II)] monohydrate] Hydrogen-bond geometry (Å, º)

**Table:**

*D*—H···*A*	*D*—H	H···*A*	*D*···*A*	*D*—H···*A*
N5—H5*A*···O1^iii^	0.89	2.20	3.0726 (18)	167
O1—H1···N2	0.87 (1)	1.99 (1)	2.8045 (19)	156 (2)

Symmetry code: (iii) −*x*+1, −*y*+1, −*z*+1.
